# Source and acquisition of rhizosphere microbes in Antarctic vascular plants

**DOI:** 10.3389/fmicb.2022.916210

**Published:** 2022-09-08

**Authors:** Sergio Guajardo-Leiva, Jaime Alarcón, Florence Gutzwiller, Jorge Gallardo-Cerda, Ian S. Acuña-Rodríguez, Marco Molina-Montenegro, Keith A. Crandall, Marcos Pérez-Losada, Eduardo Castro-Nallar

**Affiliations:** ^1^Departamento de Microbiología, Facultad de Ciencias de la Salud, Universidad de Talca, Talca, Chile; ^2^Centro de Ecología Integrativa, Universidad de Talca, Talca, Chile; ^3^Center for Bioinformatics and Integrative Biology, Facultad de Ciencias de la Vida, Universidad Andres Bello, Santiago, Chile; ^4^Laboratorio de Ecología Integrativa, Instituto de Ciencias Biológicas, Universidad de Talca, Talca, Chile; ^5^Instituto de Investigación Interdisciplinaria (I3), Universidad de Talca, Talca, Chile; ^6^Centro de Estudios Avanzados en Zonas Áridas, Facultad de Ciencias del Mar, Universidad Católica del Norte, Coquimbo, Chile; ^7^Centro de Investigación en Estudios Avanzados del Maule, Universidad Católica del Maule, Talca, Chile; ^8^Department of Biostatistics and Bioinformatics, Computational Biology Institute, George Washington University, Washington, DC, United States; ^9^Division of Emergency Medicine, Department of Pediatrics, George Washington University School of Medicine and Health Sciences, Children’s National Hospital, Washington, DC, United States; ^10^CIBIO-InBIO, Centro de Investigação em Biodiversidade e Recursos Genéticos, Universidade do Porto, Vairão, Portugal

**Keywords:** microbial ecology and diversity, plant microbiome, host microbe interactions, amplicon sequencing, South Shetland Islands, rhizosphere effects

## Abstract

Rhizosphere microbial communities exert critical roles in plant health, nutrient cycling, and soil fertility. Despite the essential functions conferred by microbes, the source and acquisition of the rhizosphere are not entirely clear. Therefore, we investigated microbial community diversity and potential source using the only two native Antarctic plants, *Deschampsia antarctica* (Da) and *Colobanthus quitensis* (Cq), as models. We interrogated rhizosphere and bulk soil microbiomes at six locations in the Byers Peninsula, Livingston Island, Antarctica, both individual plant species and their association (Da.Cq). Our results show that host plant species influenced the richness and diversity of bacterial communities in the rhizosphere. Here, the Da rhizosphere showed the lowest richness and diversity of bacteria compared to Cq and Da.Cq rhizospheres. In contrast, for rhizosphere fungal communities, plant species only influenced diversity, whereas the rhizosphere of Da exhibited higher fungal diversity than the Cq rhizosphere. Also, we found that environmental geographic pressures (i.e., sampling site, latitude, and altitude) and, to a lesser extent, biotic factors (i.e., plant species) determined the species turnover between microbial communities. Moreover, our analysis shows that the sources of the bacterial communities in the rhizosphere were local soils that contributed to homogenizing the community composition of the different plant species growing in the same sampling site. In contrast, the sources of rhizosphere fungi were local (for Da and Da.Cq) and distant soils (for Cq). Here, the host plant species have a specific effect in acquiring fungal communities to the rhizosphere. However, the contribution of unknown sources to the fungal rhizosphere (especially in Da and Da.Cq) indicates the existence of relevant stochastic processes in acquiring these microbes. Our study shows that rhizosphere microbial communities differ in their composition and diversity. These differences are explained mainly by the microbial composition of the soils that harbor them, acting together with plant species-specific effects. Both plant species acquire bacteria from local soils to form part of their rhizosphere. Seemingly, the acquisition process is more complex for fungi. We identified a significant contribution from unknown fungal sources due to stochastic processes and known sources from soils across the Byers Peninsula.

## Introduction

Microbial communities in the rhizosphere – defined as the area of soil under the biochemical influence of plant roots – play a critical role in plant health and development, soil fertility, and nutrient cycling (Philippot et al., 2013; de la Fuente Cantó et al., 2020). Rhizosphere soils are enriched in organic compounds released by plant roots, in contrast with the scarcity of organic matter in most soils, which makes them an attractive niche for microorganism colonization (Philippot et al., 2013; de la Fuente Cantó et al., 2020). The structure, diversity, and composition of microbial communities in the rhizosphere rely on soil type and edaphic properties, which interact cooperatively with the influence of plant host species through the species-specific rhizosphere effect (Berg and Smalla, 2009; Bulgarelli et al., 2013; Botnen et al., 2020; de la Fuente Cantó et al., 2020; Kumar and Dubey, 2020). Additionally to the horizontal transmission of the rhizosphere microbiome from the surrounding environment, researchers have proposed that vertical transmission of microbes from generation to generation through seeds or other propagules could be relevant in the acquisition and shape of the rhizosphere (Truyens et al., 2015; Frank et al., 2017; Vannier et al., 2018; Abdelfattah et al., 2021; Guo et al., 2021). However, data in vertical transmission studies are sparse and have reported contradictory results (Vannier et al., 2018; Abdelfattah et al., 2021; Guo et al., 2021).

Plant roots secrete many compounds, including ions (organic and inorganic), phytosiderophores, polysaccharides, vitamins, amino acids, nitrogenated bases, and nucleosides (Bulgarelli et al., 2013; Philippot et al., 2013; Matilla and Krell, 2018). These rhizodeposits change the soil’s biological, chemical, and physical conditions and account for 11% of the photosynthetically fixed carbon and 10–16% of total plant nitrogen (Jones et al., 2009; Bulgarelli et al., 2013; Dotaniya and Meena, 2015). Therefore, plants act as ecosystem engineers by changing the soil’s redox potential, pH, aggregation, and water–nutrient availability (de la Fuente Cantó et al., 2020).

Many studies have suggested that root exudates differ between plant species, genotypes, and phenological status (Dennis et al., 2010; Matilla and Krell, 2018; de la Fuente Cantó et al., 2020; Vieira et al., 2020). The different composition of the exudates generates a species-specific or genotype-specific rhizosphere effect that exerts selective pressures on the composition of the rhizosphere microbial communities (Dennis et al., 2010; Matilla and Krell, 2018; de la Fuente Cantó et al., 2020; Vieira et al., 2020). Although evidence indicates that host species and genotypes can induce a selective influence on specific microbial taxa such as mycorrhizal fungi and some nitrogen-fixing bacteria (e.g., *Rhizobium*), its effect has a weaker, broader impact on microbial community composition than soil abiotic conditions (Bonito et al., 2014; Fierer, 2017; Yeoh et al., 2017; Thiergart et al., 2020; Vieira et al., 2020). Recent studies have shown that the effect of soil’s abiotic conditions outweighs the impact of the genotype or plant species. For example, the variance in rhizosphere microbial communities across members of a specific plant species grown in different soil types is usually more significant than the variance observed between different plant species grown in the same soil (Bonito et al., 2014; Yeoh et al., 2017; de la Fuente Cantó et al., 2020; Vieira et al., 2020).

In addition to these deterministic factors, soil microbial communities can also be affected by stochastic processes that generate species compositional patterns indistinguishable from those produced at random (Tripathi et al., 2018; Richter-Heitmann et al., 2020; Hussain et al., 2021; Zhang et al., 2021). These stochastic processes involve random proliferation, death, and dispersal events (Tripathi et al., 2018; Richter-Heitmann et al., 2020; Hussain et al., 2021; Zhang et al., 2021). Then, the ratio between stochastic and deterministic effects on microbial communities affects the species diversity and composition over temporal and spatial scales (Tripathi et al., 2018; Richter-Heitmann et al., 2020).

There is broad agreement that beneficial plant–microorganism interactions in roots facilitate the adaptation of these sessile organisms to a changing environment (Dimkpa et al., 2009; Hayat et al., 2010; Bulgarelli et al., 2013; Matilla and Krell, 2018; de la Fuente Cantó et al., 2020). Beneficial root-associated microorganisms can directly affect plant growth and development by multiple mechanisms such as nitrogen fixation and metabolism, nutrient solubilization, and the production of phytohormones and volatile compounds (Dimkpa et al., 2009; Bulgarelli et al., 2013; Imam et al., 2016; Matilla and Krell, 2018; Vishwakarma et al., 2020). Additionally, rhizosphere microorganisms can also enhance plant fitness by different strategies such as protection against pathogens (i.e., triggering defense responses in the plant, producing antimicrobials, or competing with pathogens for nutrients and ecological niches) and increasing plant stress tolerance (i.e., heavy metal sequestration, osmotic regulation, and tolerance to drought and high salinity) (Bulgarelli et al., 2013; Imam et al., 2016; Matilla and Krell, 2018; Molina-Montenegro et al., 2019, 2020; Vishwakarma et al., 2020). These beneficial plant–microbe interactions increase the plant foraging capacity and stress resistance, where microbes confer new or redundant functions to the plant host and act as biocontrol agents against pathogens.

Beneficial plant–microbe interactions are especially relevant in extreme environments (Rodriguez et al., 2008; Newsham, 2011; Acuña-Rodríguez et al., 2020), where only a few organisms can thrive in conditions approaching life’s limits, such as those in Antarctica. Moreover, the harsh conditions of Antarctica and their relative geographical isolation have only allowed the colonization of ice-free zones by two vascular plants, *Colobanthus quitensis* (Cq, Caryophyllaceae) and *Deschampsia antarctica* (Da, Poaceae) (Moore, 1970). These conditions make Antarctica a natural laboratory where it is possible to study plant–microbe interactions in a pristine and streamlined environment compared to other natural habitats.

Although both plants are present in maritime Antarctica, Da is mainly observed in more stressful abiotic conditions (Atala et al., 2019; Molina-Montenegro et al., 2019). Cq, for its part, usually grows in sites under more mild abiotic conditions (Atala et al., 2019; Molina-Montenegro et al., 2019). However, Da can act as a nurse species for Cq, allowing it to grow in less favorable environmental conditions by forming tussocks (Atala et al., 2019; Molina-Montenegro et al., 2019). Physiological experiments have demonstrated that rhizosphere microorganisms may have an active role in the performance of Da and Cq against environmental stressors that naturally occur in maritime Antarctica, such as high salinity, UV-B radiation, and increases in atmospheric temperature (Gallardo-Cerda et al., 2018; Ramos et al., 2018; Ballesteros et al., 2020). However, microbial communities in the rhizosphere of these plants have not been extensively studied, and the few existing studies have used small sample sizes. Early works used culture-dependent and molecular markers (16S rRNA DGGE and microarrays) to characterize rhizosphere bacteria showing that Da was dominated by psychrotolerant representatives of *Pseudomonas*, *Flavobacterium*, and *Arthrobacter* (Barrientos-Díaz et al., 2008). Then, the use of 16S rRNA showed that bacteria found in the rhizosphere of Da and Cq were very different from those reported in adjacent bulk soil (Teixeira et al., 2010, 2013). Additionally, no differences were found between the bacterial communities of Da and Cq, and both rhizospheres were composed of Firmicutes, Actinobacteria, and Proteobacteria (Teixeira et al., 2010, 2013; Jorquera et al., 2016; Rabert et al., 2020; Znój et al., 2021). One of the most recent studies used shotgun sequencing to demonstrate that Da and Cq rhizospheres were composed mainly of Proteobacteria, Actinobacteria, and Bacteroidetes, finding differences between the rhizosphere bacteria of both plants only at the genus level (Molina-Montenegro et al., 2019).

In this study, we investigated bacterial and fungal community composition and structure (16 rRNA and ITS1 amplicon sequencing) using the only two native Antarctic plants, *Deschampsia antarctica* (Da) and *Colobanthus quitensis* (Cq), as models. We tested whether the rhizosphere is significantly different in diversity and composition from other compartments and whether microbes come from local soils or other sources. We interrogated bulk soil, rhizosphere-surrounding soil (RSS), and rhizosphere microbial communities at six locations in the Byers Peninsula, Livingston Island, Antarctica, using individual plant species and their association (Da.Cq).

## Materials and methods

### Site description and soil sample processing

**FIGURE 1:**
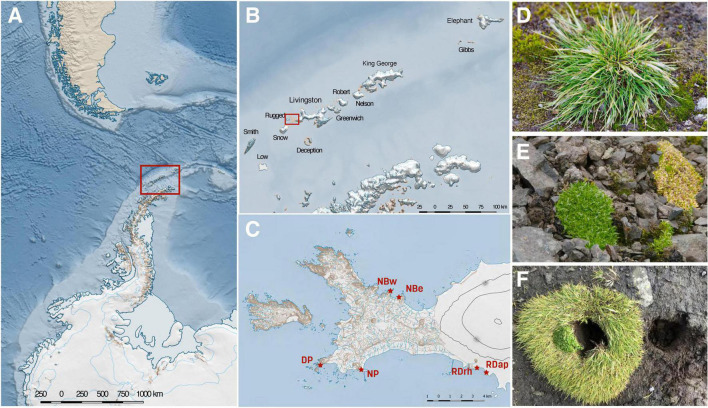
Map and description of the study sites. The six sampling sites are located in the Byers Peninsula, Livingston Island, Antarctica. The study site **(A)** is located in the maritime Antarctica, specifically in Livingston Island **(B)**. The Byers Peninsula is devoid of ice during the Austral Summer **(C)**, which favors the growth of both *Deschampsia antarctica* [Da] **(D)** and *Colobanthus quitensis* [Cq] **(E)**. In some instances, *C. quitensis* grows in association with *D. antarctica* [Da.Cq] **(F)**. NBw and NBe = North Beach West and East, respectively; DP and NP = Devils Point and Nikopol Point, respectively; and RDrh and RDap = Rotch Dome Ritli Hill and Rotch Dome Amadok Point, respectively.

We collected samples during the growing season in the Austral Summer (February) of 2018 as part of the 55th Scientific Antarctic Expedition (ECA-56). Rhizosphere (51 samples) and bulk soil (45 samples) were collected from six sampling sites (Devil’s Point [DP], North Beach West [NBw], North Beach East [NBe], Nikopol Point [NP], Rotch Dome Ritli Hill [RDrh], and Rotch Dome Amadok Point [RDap]) at the Byers Peninsula, Livingston Island, Antarctica (Figure 1 and Supplementary Table 1).

Rhizosphere soils of *Deschampsia antarctica* (Da, 25 samples), *Colobanthus quitensis* (Cq, 13 Samples), and Cq growing in association with Da (Da.Cq, 13 samples) were sampled at nearly sea level. Each sampling site harbors several biological replicates from bulk soil and at least one plant species, within a ≤ 5 m radius. For each type of sample (plant attached soil or bulk soil), we take a soil volume of 0.5 L in sterile polypropylene bags. We use the term compartment to refer to the different soil fractions from now on. The bulk soil compartment corresponds to soil free of plants, mosses, lichens, or evident organic matter in the vicinity of each plant or plant group in a radius no greater than 1.5 m. The soil attached to the plants was divided into two fractions (compartments). The first corresponds to the rhizosphere-surrounding soil (RSS), the soil fraction released by handshaking the roots. The second fraction corresponds to the rhizosphere, which is the soil firmly adhered to the roots (2–5 mm thick layer on the surface of the roots). For each sample and compartment, fine soil particles (≤2 mm) were carefully separated from pebbles and roots using a sterile 2 mm metal mesh and stored in 50 mL sterile conical tubes at −20°C until DNA extraction. All samples were processed in a biosafety level 2 hood, and plant and soil samples were collected with the permission of the Chilean Antarctic Institute (INACH).

### DNA extraction and amplicon sequencing

According to the manufacturer’s instructions, DNA was extracted from 0.5 to 2 g of soil using a QIAamp PowerFecal DNA Kit (Qiagen, Düsseldorf, Germany). DNA was quantified using the Qubit DNA probe (Invitrogen), quality was assessed by spectrophotometry (A260:A280 ratio), and DNA integrity by agarose gel electrophoresis. For sequencing, we targeted the V4 region of the 16S rRNA gene using the 515F (Parada) 5′-GTGYCAGCMGCCGCGGTAA-3′ and 806R (Apprill) 5′-GGACTACNVGGGTWTCTAAT-3 primers and the ITS1 gene using the ITS1F 5′-CTTGGTCATTTAGAGGAAGTAA-3′ and ITS2 5′-GCTGCGTTCTTCATCGATGC-3′ (The Earth Microbiome Project Consortium et al., 2017). Sequencing was conducted on a MiSeq Illumina sequencer (MiSeq Reagent Kit v2, 2 × 150 bp for 16S rRNA and 2 × 250 bp for ITS) at the Argonne National Laboratory (Lemont, IL, United States).

### Amplicon sequence analysis

Sixteen-S and ITS pair-end reads were demultiplexed and analyzed using amplicon sequence variants (ASVs) as implemented in “DADA2 v1.8” (Callahan et al., 2016). Due to the variable lengths of the ITS reads, we used different settings for the trimming and filtering steps of 16S rRNA and ITS sequences using Cutadapt (Martin, 2011). The following filtering parameters were used for 16S rRNA and ITS sequences (maxN = 0, maxEE = c(2,2), and truncQ = 2). Specific trimming parameters for 16S rRNA reads were truncLenc (240,180) and for ITS sequences minLen = 50. For error rate learning, dereplication, denoising, and merging steps, we followed the DADA2 Pipeline Tutorial (1.16) https://benjjneb.github.io/dada2/tutorial.html and the DADA2 ITS Pipeline Workflow (1.8) https://benjjneb.github.io/dada2/ITS_workflow.html for 16S rRNA and ITS, respectively. Briefly, the error rate (“learnErrors” command) was estimated for the quality-filtered reads followed by a dereplication step (“derepFastq” command) and denoising (“dada” command, using the error rate from the step above). Finally, reads pairs were merged using the “mergePairs” command.

After building the ASVs tables (“makeSequenceTable” command) and removing chimeras (“removeBimeraDenovo” command), each ASV was taxonomically assigned using Silva database v132 (Quast et al., 2012) for bacteria and full UNITE + INSD (v8.0) for fungi (UNITE Community, 2019).

Next, we inferred phylogenetic trees using the maximum likelihood optimality criterion implemented in “FastTree v2.1.10” (Price et al., 2010). Finally, ASVs that were not assigned to bacteria or fungi (including 61 ASVs assigned to Archaea) were removed, and samples with less than 1,000 reads were discarded.

### Community richness and diversity among plant species and compartments

Alpha diversity metrics were calculated using the R packages phyloseq v1.34.0 (Chao1 and Shannon indexes) (McMurdie and Holmes, 2013) and btools v0.0.1^1^ for Faith’s phylogenetic diversity index (PD).

The distribution of alpha diversity (Chao1, Shannon, and PD) across predictors (plant species, compartment, and sampling sites) was visualized using boxplots (plot_alpha function) as in the microeco package (Liu et al., 2021). The statistical significance of the predictors was tested using an analysis of variance (ANOVA) using a two-factor interaction (three-factor interaction did not render statistically significant results) and pairwise comparisons with Tukey’s HDS.

Beta diversity metrics (Bray–Curtis and weighted UniFrac) for bacterial and fungal communities were calculated using the “phyloseq v1.34.0” package. To identify drivers of beta diversity, we first tested whether the dispersion among groups was homogeneous using the function betadisper implemented in the “vegan v2.5.7” package (Oksanen et al., 2020). Then, we assessed the statistical significance of the beta diversity among sampling sites, plant species, and compartments using a W_d_* test paired with W_d_* *post-hoc* test (Hamidi et al., 2019). Additionally, we also quantified the proportion of the variance explained by the variables sampling sites, plant species, and compartments, by performing a variance partitioning analysis (VPA) as implemented in the varpart function in the “vegan v2.5.7” package (Oksanen et al., 2020).

We performed a constrained ordination analysis to extract and summarize the sample variation that the set of explanatory variables can explain. To decide whether to apply the linear or unimodal ordination method, we first calculated a detrended correspondence analysis (DCA) using the decorana function in the “vegan v2.5.7” package (Ozimek and Hanaka, 2020). Then, we carried out a redundancy analysis (RDA) using the Hellinger-transformed Bray–Curtis and weighted UniFrac distances based on the ASV abundance matrix for bacterial and fungal communities in “Ampvis2 v2.7.6” (Kasper et al., 2018). To avoid overfitting, we reduced the number of explanatory variables using a forward variable selection procedure as implemented in the ordiR2step function in the “vegan v2.5.7” package (Ozimek and Hanaka, 2020). The statistical significance of the selected variables was quantified by an ANOVA test with Holm correction for multiple testing. Statistical significance of the RDAs was tested using 9999 permutations.

### Assessment of microbial community composition

We constructed heatmaps of the most relative abundant taxa (>1%) agglomerated at the genus or the best taxonomic hit level to assess the taxonomic composition for bacterial and fungal communities “Ampvis2 v2.7.6” package (Kasper et al., 2018). To differentiate microbial markers in each plant species (rhizosphere and RSS) and bulk soil, we used a random forest classification model implemented in the trans_diff function (method = “rf”) in the “microeco v0.6.0” package (Liu et al., 2021) on taxa that were present in at least 60% of the samples. Microbial markers were selected based on their importance (genera above the average of the mean decrease in the Gini coefficient for fungi and the top ten for bacteria) and statistical significance (*p*-value < 0.05).

To visualize unique and shared ASVs between rhizospheres of the different plant species in the different sampling sites, we used Venn diagram analysis implemented in R package “microeco v0.6.0” (Liu et al., 2021).

### Inferring the potential source of rhizosphere microbial communities

To determine the potential source of bacteria and fungi in the rhizosphere of Da, Cq, and Da.Cq plants, we used the “SourceTracker” software package (Knights et al., 2011) using Devil’s Point as a model site. SourceTracker is a Bayesian approach to identifying microbial sources and their proportions in amplicon data (Knights et al., 2011). This approach models a sink sample as a mixture of sources where the mixing proportions are unknown (Knights et al., 2011).

Here, we used the rhizosphere samples of Da, Cq, and Da.Cq from Devil’s Point as sink samples and examined potential source samples that included distant rhizospheres, RSS, and bulk soil sampled at Byers sites other than DP. Likewise, we added local source samples corresponding to rhizospheres, RSS, and bulk soil from DP. The part of the community that does not match any source sample was assigned to an “unknown” source.

### Data availability

Raw sequences are publicly available under NCBI SRA BioProject PRJNA818311.

## Results

### Microbial diversity in antarctic vascular plants

To understand and analyze the microbial communities in the rhizosphere of Antarctic vascular plants, we used 16S rRNA and ITS amplicon sequencing in rhizosphere soil samples from *D. antarctica*, *C. quitensis*, and their association (Da.Cq), growing at six locations in the Byers Peninsula, Livingston Island, Antarctica (Figure 1). A total of 25 samples of Da, 14 samples of Cq, 14 samples of Da.Cq, and 45 bulk soil samples were collected during the growing season in February 2018, resulting in 145 and 146 16S rRNA and ITS libraries, respectively. Detailed information about the samples is available in Supplementary Table 1.

First, we interrogated our data to assess whether microbial communities were comparable regarding their members and their corresponding distribution among species (Da, Cq, and Da.Cq) and compartments (rhizosphere, RSS, and bulk soil) for the different locations (DP, NBw, NBe, NP, RDrh, and RDap). We found significant differences in richness (Chao 1) and diversity (Shannon and PD) among bacterial communities from the different plant species, compartments, and sampling sites (ANOVA, *p*-value ≤ 0.05, Supplementary Table 3). Conversely, fungal communities exhibited differences in richness among compartments and sampling sites but not among different plant species (ANOVA, *p*-value ≤ 0.05, Supplementary Table 3). In turn, the evenness and phylogenetic diversity (Shannon and PD) of fungi were significantly different between plant species and sampling sites, but not between compartments (ANOVA, *p*-value ≤ 0.05, Supplementary Table 3).

We then conducted *post-hoc* tests to identify potential differences in richness and diversity among soil compartments (rhizosphere, RSS, and bulk soil). Bacterial richness and diversity (Chao1, Shannon and PD) were significantly higher (Tukey’s HDS, *p*-value ≤ 0.05, Supplementary Table 3) in RSS compared to the rhizosphere. However, we did not find differences between RSS and bulk soil or rhizosphere and bulk soil (Supplementary Figure 1). For fungi, we only found that RSS presented a higher richness (Chao1) than the rhizosphere (Tukey’s HDS, *p*-value ≤ 0.05, Supplementary Table 3). However, we did not find significant differences in terms of diversity (Shannon and PD) (Supplementary Figure 1).

**FIGURE 2:**
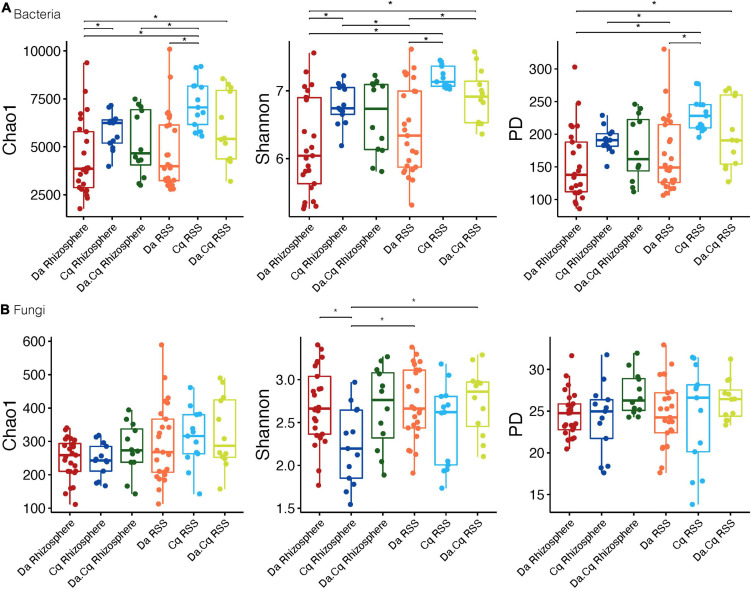
Bacteria **(A)** and fungi **(B)** alpha diversity (Chao1; Shannon; phylogenetic diversity) for plant species and their compartments (rhizosphere, rhizosphere-surrounding soil [RSS]) *Deschampsia antarctica* [Da], *Colobanthus quitensis* [Cq], and their association [Da.Cq]. Dots represent data points, and boxplots represent the interquartile range of alpha diversity. Statistically significant differences (Tukey’s HSD *p*-value ≤ 0.05, for predicted alpha diversity by a linear model) are marked by brackets.

For plants, we found that bacterial richness and diversity (Chao1, Shannon, and PD) in Cq plants (rhizosphere and RSS) were higher (Tukey’s HDS, *p*-value ≤ 0.05, Supplementary Table 3) than in Da plants (rhizosphere) (Figure 2). Likewise, bacterial communities of Da.Cq RSS showed higher richness and diversity than those in the Da rhizosphere (Tukey’s HDS, *p*-value ≤ 0.05, Supplementary Table 3). Conversely, we found that the diversity (Shannon) of fungi in Da plants (rhizosphere and RSS) was significantly higher than the fungal diversity of Cq rhizosphere (Tukey’s HDS, *p*-value ≤ 0.05). Additionally, fungal communities of Da.Cq RSS were significantly (Tukey’s HDS, *p*-value ≤ 0.05, Supplementary Table 3) more diverse (Shannon) than the ones in the Cq rhizosphere (Figure 2).

**FIGURE 3:**
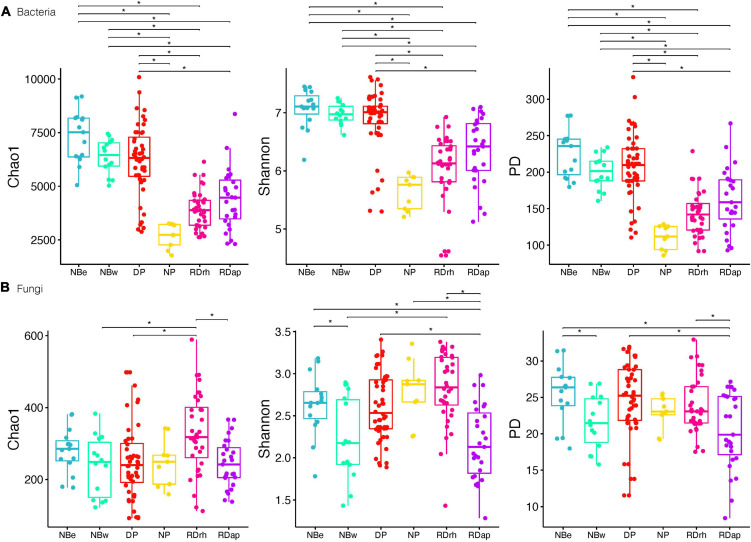
Bacteria **(A)** and fungi **(B)** alpha diversity (Chao1; Shannon; phylogenetic diversity) for sampling sites North Beach East [NBe], North Beach West [NBw], Devil’s Point [DP], Nikopol Point [NP], Rotch Dome Ritli Hill [RDrh], and Rotch Dome Amadok Point [RDap]. Dots represent data points, and boxplots represent the interquartile range of alpha diversity. Statistically significant differences (Tukey’s HSD *p*-value ≤ 0.05, for predicted alpha diversity by a linear model) are marked by brackets.

Finally, we found that the different sampling sites (Figure 3) can be divided according to their bacterial diversity and richness (Tukey’s HDS, *p*-value ≤ 0.05, Supplementary Table 3): sampling sites of high diversity (Shannon and PD) and richness (Chao1), such as NBe, NBw, and DP, and sampling sites of low diversity and richness, such as RDap, RDrh, and NP. Conversely, there were no apparent differences between the richness and diversity of the different sampling sites for fungal communities. For example, some sampling sites like RDrh presented a higher richness (Tukey’s HDS, *p*-value ≤ 0.05, Supplementary Table 3) than RDap, NBw, and DP (Figure 3). Moreover, sites such as RDap (Figure 3) presented a lower diversity (Tukey’s HDS, *p*-value ≤ 0.05, Supplementary Table 3) compared to the sites DP, NBe (Shannon and PD), and RDrh (Shannon).

These results suggest that bacterial alpha diversity was higher in RSS than in plants’ rhizospheres. For instance, bacterial diversity and richness were higher overall in the Cq plants (rhizosphere and RSS) compared to Da plants, with the diversity and richness levels of the Da.Cq bacterial communities intermediate with those observed for Da and Cq individually (although the latter was not statistically significant). This pattern splits the sampling locations into two groups: high diversity and richness sites (Nbe, NBw, and DP) and low diversity and richness sites (NP, Rdap, and RDrh). Fungal communities, in turn, do not show a clear pattern of diversity or richness among locations. As opposed to bacterial richness and diversity, rhizosphere and RSS in Da plants exhibited more diverse fungi than Cq, with RSS being the most diverse compartment.

### Drivers of microbial community structure

Next, we wanted to assess whether microbial communities were homogeneous or presented a high turnover among locations, plant species, or compartments. For this, we calculated two beta diversity indices (Bray–Curtis and weighted UniFrac) and found that our data rejected the hypothesis of homogeneity of variance (ANOVA, *p*-value ≤ 0.0001). Using a robust test, we found statistically significant differences (W_d_* test, *p*-value ≤ 0.0001) between beta diversity (Bray–Curtis and weighted UniFrac) of microbial communities from different sampling sites and compartments by plant species (species compartments; Supplementary Table 4). Pairwise comparisons between sampling sites showed statistically significant differences in beta diversity of bacteria and fungi (Bray–Curtis and weighted UniFrac) between all sampling sites (W_d_* test, *p*-value ≤ 0.007, Supplementary Table 4). Likewise, there were statistically significant differences (W_d_* test, *p*-value ≤ 0.036, Supplementary Table 4) in beta diversity of bacteria (Bray–Curtis and weighted UniFrac) from all plant species compartments, with some exceptions when Bray–Curtis dissimilarity was used, such as in rhizospheres of Da vs. Da.Cq (W_d_* test, *p*-value = 0.065) or between the rhizosphere of each plant and its RSS counterpart (W_d_* test, *p*-value ≥ 0.185). For fungi, we found that most plants’ community composition and compartments presented statistically significant differences (W_d_* test, *p*-value ≤ 0.036). The exceptions were the Cq rhizosphere vs. Cq RSS; Da RSS vs. Da.Cq RSS; Da.Cq rhizosphere vs. bulk soil, Da rhizosphere, and Da.Cq RSS (weighted UniFrac only); Da rhizosphere (weighted UniFrac only) vs. Da RSS and Da.Cq RSS. These results suggest that bacterial species’ turnover is significantly different among sampling sites and different compartments by plant species. However, for fungi, species turnover was significant between different sampling sites and between specific compartments by plant species, such as the Da and Cq rhizospheres and between these rhizospheres and the bulk soil.

We used a variance partitioning analysis (VPA) to quantify the variation in microbial composition explained by plant species compartments, sampling sites, and the shared variation explained by both factors (Supplementary Table 4). This analysis showed that the highest variance in the composition of the microbial communities was given by the sampling site (21.58/26.63%) for bacteria and (6.18/19.55%) for fungi (for Bray–Curtis and weighted UniFrac, respectively). Likewise, plant species contributed 6.26/10.71% of the variance in bacteria and 1.96/4.1% in fungi (Bray–Curtis and weighted UniFrac, respectively). Both variables explained 31.33/43.27% of the variance in bacteria and 8.92/27.66% in fungi (including the shared variance). Other unaccounted variables influence the remaining variance.

To further explore community structure in our dataset, we performed a constrained ordination analysis extracting and summarizing the maximum variation of the microbial composition explained by statistically significant ecological variables recorded in our sampling. First, using detrended correspondence analysis (DCA), we determined the suitability of a linear ordination method (first DCA axis < 3); hence, a redundancy analysis (RDA) was performed. Then, we performed a forward selection procedure for explanatory variables to reduce the number of explanatory variables entering the RDA while keeping the variation explained by them to the maximum. For bacteria (Bray–Curtis and weighted UniFrac), the multivariate space was constrained by sampling site, plant species compartment, and latitude. In contrast, for fungi, the constrained variables were sampling site (Bray–Curtis and weighted UniFrac), compartment (Bray–Curtis), longitude (Bray–Curtis), plant species (weighted UniFrac), and latitude (weighted UniFrac).

**FIGURE 4:**
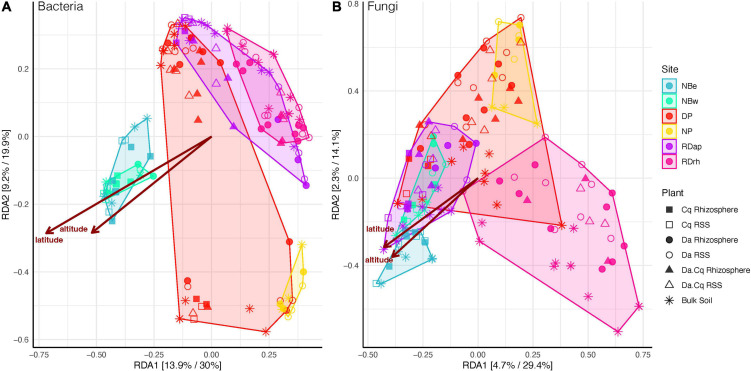
Bacteria **(A)** and fungi **(B)** beta diversity for *Deschampsia antarctica* [Da], *Colobanthus quitensis* [Cq], and their association [DaCq]. A redundancy analysis of Hellinger-transformed weighted UniFrac distance was statistically chosen to describe microbial community structure in a supervised approach. Each axis shows the percentage of variance explained in an unsupervised and supervised analysis.

The RDA (Figure 4 and Supplementary Figure 2) showed that the constrained space explained 44 and 15% of the total variance for bacteria and fungi, respectively. The first two axes explained approximately 50% of the constrained variance for bacteria and 45% for fungi (using both weighted UniFrac and Bray–Curtis) within the total constrained space. The RDA emphasizes the relevance of geographic factors such as the sampling site, latitude, longitude, and altitude of the samples as drivers of dissimilarities in the turnover of microbial communities. This pattern becomes evident when observing how the samples from the beaches north of the peninsula (NBe and NBw) are somewhat separated from the rest of the samples, directly influenced by their latitude and altitude (see Supplementary Table 1). In contrast, fungal communities were structured differently than bacteria, suggesting that other rhizosphere acquisition and source modes govern their structure and composition.

### Taxonomic composition of plant’s rhizospheres and soil microbial communities

**FIGURE 5:**
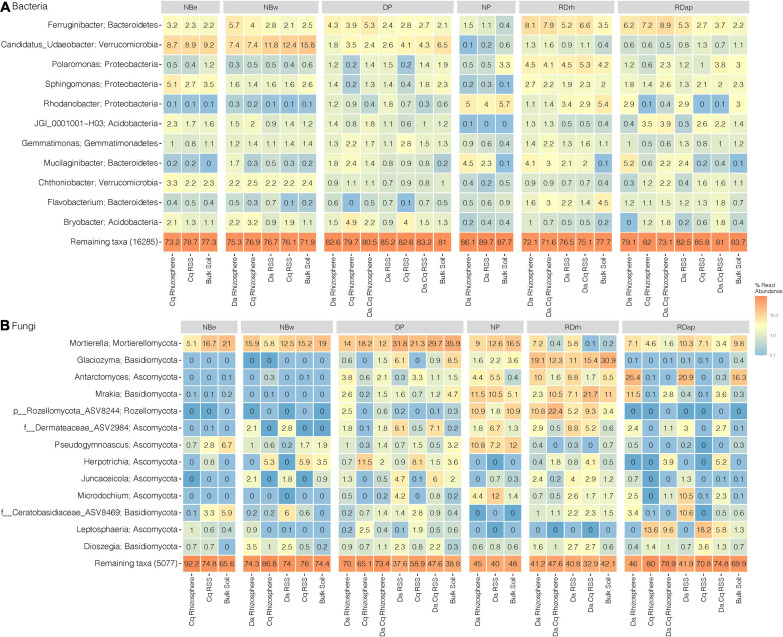
Microbial community composition for bacteria **(A)** and fungi **(B)** in *Deschampsia antarctica* (Da), *Colobanthus quitensis*(Cq), and their association (DaCq). We took only the most abundant taxa (abundance ≥ 1% in the total samples) at their genus or best-hit taxonomic classification by sampling site and plant species compartment. Heatmap colors represent the taxa relative abundance (arithmetic mean by plant compartment) on a logarithmic scale.

To explore the taxonomic composition of bulk soils and plant’s rhizospheres RSSs in each sampling site, we generated a heatmap of the most abundant taxa (abundance ≥ 1% in the total samples) at the genus or best-hit taxonomic classification (Figure 5). Only 11 genera in bacteria and 13 genera in fungi presented a relative abundance ≥ 1% in the total samples (Figure 5). The most abundant bacterial genera belonged to the Bacteroidetes (*Ferruginibacter*, *Mucilaginibacter*, and *Flavobacterium*), Proteobacteria (*Polaromonas*, *Sphingomonas*, and *Rhodanobacter*), Verrucomicrobia (*Candidatus Udaeobacter* and *Chthoniobacter*), Acidobacteria (Acidobacteria bacterium JGI0001001-H03 and *Bryobacter*), and Gemmatimonadetes (*Gemmatimonas*) phyla (Figure 5A). However, at higher taxonomic levels, the most abundant phyla were Proteobacteria, Bacteroidetes, Acidobacteria, Verrucomicrobia, and Actinobacteria, representing 75–92% of the total abundance of ASVs in the different compartments and sampling sites (Figure 5A). For fungi, the most abundant genera belonged to Mortierellomycota (*Mortierella*), Basidiomycota (*Glaciozyma*, *Mrakia*, Ceratobasidiaceae-ASV8469, and *Dioszegia*), Ascomycota (*Antarctomyces*, Dermateaceae-ASV2984, *Pseudogymnoascus*, *Herpotrichia*, *Juncaceicola*, *Microdochium*, and *Leptosphaeria*), and the Rozellomycota (ASV8244) phyla (Figure 5B). These four phyla accounted for 57–100% of the total abundance of ASVs in the different compartments at each sampling site (Figure 5B).

Next, we used a random forest classification model to statistically determine significant differences between taxa present in the microbial communities of the rhizosphere and the RSS of the different plant species and the bulk soil (Supplementary Figure 3). We found that bacteria belonging to the *Dyadobacter*, *Mucilaginibacter, and Candidatus Xiphinematobacter* genera were consistently more abundant (*p*-value ≤ 0.05) in the sections associated with plants (rhizosphere and RSS) than in bulk soil. Moreover, these genera formed an abundance gradient from the rhizosphere to the bulk soil with RSS in between, and another gradient between Da and Cq with Da.Cq in between. Likewise, bacterial ASVs assigned to *Devosia*, *Galbitalea*, *Lysinomonas*, *Asticcacaulis*, *Prosthecobacter*, and *Neorhizobium* (the latter especially abundant in Da) showed a higher abundance in the compartments of the plants than in the bulk soil (*p*-value ≤ 0.05). Conversely, bacterial ASVs assigned to the genus *Pseudarthrobacter* were more abundant in bulk soil and RSS than in rhizospheres (bulk soil > Da RSS > Da.Cq RSS > Cq RSS).

For fungi, we found two genera, *Dioszegia* and *Cheilymenia*, more abundant in plant compartments (rhizosphere and RSS) than in bulk soil (Supplementary Figure 3, *p*-value < 0.05). In both plants and their association (Da.Cq), *Dioszegia* was more abundant in the RSS than in the rhizosphere. At the same time, *Cheilymenia* was more abundant in the rhizosphere than in the RSS (Supplementary Figure 3, *p*-value < 0.05). Other genera such as *Antarctomyces*, *Glaciozyma*, *Mrakia*, and *Juncaceicola* were abundant in both the bulk soils and plant compartments (rhizosphere and RSS) of Da and Da.Cq, but were almost absent in the rhizosphere and the RSS of Cq (Supplementary Figure 3, *p*-value ≤ 0.05). Conversely, the genera *Leptosphaeria* and *Verrucaria* were more abundant in Cq plants (rhizosphere and RSS) than in Da and Da.Cq plants (Supplementary Figure 3, *p*-value ≤ 0.05). Finally, the genus *Microdochium* was more abundant in Da and Da.Cq plants (rhizosphere and RSS) than in Cq plants and bulk soils (Supplementary Figure 3, *p*-value ≤ 0.05).

Altogether, these results show that there are taxa from both bacteria and fungi, whose abundances are significantly different among the studied compartments (rhizosphere, RSS, and bulk soil) and among different plants, which is especially marked in the case of fungi. Furthermore, these results suggest that while soil microbes are also present in RSS and rhizosphere soil, plants can select and foster specific microbes, forming distinguishable and detectable gradients.

### Core microbiome and source of the rhizosphere microbial communities

Finally, we wanted to assess to what extent microbes are shared and potentially linked in source among the rhizosphere of Da and Cq. We constructed Venn diagrams to determine shared and unique (100% of prevalence) ASVs from bacteria and fungi between rhizospheres of the same plant species in different sampling sites regardless of their relative abundance. Our results show that few bacteria and fungi ASVs in each plant’s rhizospheres were shared (100% of prevalence) between all sampling sites (Supplementary Figures 4, 5). For Da, only 4.7% (827 ASVs) of bacterial ASVs and 0.3% (7 ASVs) of fungal ASVs were shared (100% of prevalence) between the six sampling sites, where Devil’s Point [28.1% (4974 ASVs) and 26.9% (558 ASVs) for bacteria and fungi, respectively] and Rotch Dome Ritli Hill [6.9% (1228 ASVs) and 28% (581 ASVs) for bacteria and fungi, respectively] having a significant number of exclusive ASVs. For Cq, 6.9% (1032 ASVs) of bacteria and 1% (14 ASVs) of fungi ASVs were shared (100% of prevalence) between the four sampling sites, with North Beach East (18.3%/2749 ASVs) and Rotch Dome Amadok Point (12.9%/1945 ASVs) concentrating most of the exclusive bacteria ASVs. For fungi in Cq’s rhizosphere, the four sites showed many exclusive ASVs ranging from 15.8 (220 ASVs) in Devil’s Point to 27.8% (387 ASVs) in North Beach East. Finally, for Da.Cq, we found that 15.1% (2175 ASVs) of bacteria ASVs and 1.8% (26 ASVs) of fungi ASVs were shared (100% of prevalence) between the three sampling sites. For bacteria, Devil’s Point showed most of the exclusive ASVs (48.5%/6996 ASVs), while for fungi, Devil’s Point and Rotch Dome Ritli Hill accounted for 39.4% (561 ASVs) and 30.4% (432 ASVs) of the exclusive ASVs, respectively. These results suggest a low number of microbial ASVs were prevalent in all rhizospheres of plants from the same species (i.e., Da, Cq, and Da.Cq) growing in different sampling sites across Byers. There is also a gradient in the size of the core microbiome between the different plants, being more numerous in Da.Cq followed by Cq and Da (Da.Cq > Cq > Da). Finally, sites such as Devil’s Point presented the highest number of ASVs exclusive to both bacteria and fungi in the rhizosphere of Da and Da.Cq, being Cq the exception.

**FIGURE 6:**
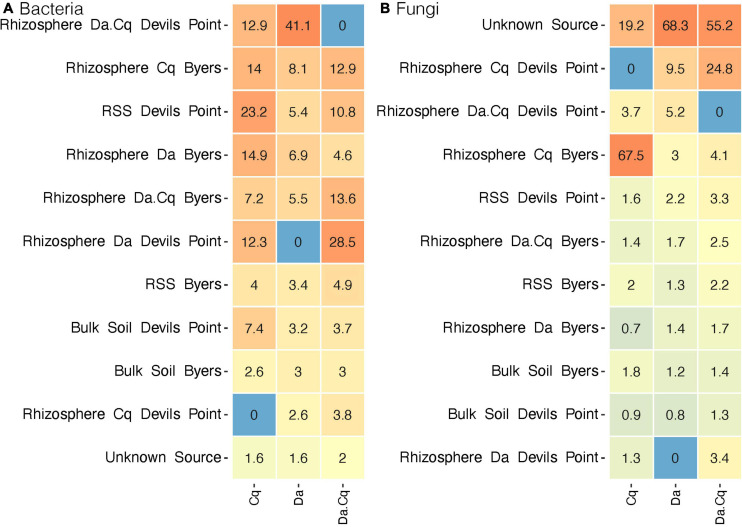
SourceTracker analyses for bacteria **(A)** and fungi **(B)** in the rhizosphere of *Deschampsia antarctica* [Da], *Colobanthus quitensis* [Cq], and their association [DaCq]. Rhizosphere samples collected at Devil’s Point were considered sinks and samples from other sites in Byers as potential sources for bacteria and fungi. Heatmap colors represent the relative contribution of each source to the plant’s rhizospheres composition on a logarithmic scale.

We then set out to quantify the influence of different microbial sources on the taxonomic composition of the rhizosphere communities by plant species. First, we conducted a source tracking analysis in the rhizosphere samples collected in Devil’s Point as a model site (Figure 6). Our analysis indicates that bacterial ASVs from local sources (i.e., within DP) were the most common source of the Da, Cq, and Da.Cq rhizospheres in Devil’s Point. Specifically, for Cq plants, RSS from DP was the primary source of rhizosphere bacteria, followed by Da rhizosphere from other sampling sites and the rhizosphere of Da.Cq from DP. In the case of the rhizosphere of Da plants, the Da.Cq rhizosphere from DP was the primary source of bacteria, followed by Cq and Da rhizospheres from other Byers’ sampling sites. Finally, for the rhizosphere of Da.Cq plants, the Da rhizosphere from DP was the primary source of bacteria, followed by Da.Cq and Cq rhizospheres from other Byers’ sampling sites.

For fungal communities, the source tracking analysis (Figure 6) revealed that most fungi ASVs in the rhizospheres of Da and Da.Cq came from unknown sources (sources for which there is no data collected since they were present only in the sink plant’s rhizospheres). In contrast, the source of the fungal communities from the Cq rhizosphere was found mainly in the Cq rhizospheres from other Byers’ sampling sites. However, for Da, we found a small contribution of Cq and Da.Cq fungi from local rhizospheres in DP. Likewise, for Da.Cq plants in DP, we found a small contribution of local rhizospheres from Devil’s Point Da and Cq rhizospheres and Cq rhizospheres from other sampling sites in Byers.

Taken together, we showed the bacterial communities in the rhizosphere of *D. antarctica*, *C. quitensis*, and their association Da.Cq mainly were composed of site-specific ASVs. Nevertheless, these ASVs were shared between the rhizospheres of the different plant species, especially between Da and Da.Cq. Likewise, the fungal communities in the rhizosphere of *D. antarctica* and Da.Cq were also made up of site-specific ASVs. This trait is more potent than that observed in bacteria except for *C. quitensis*, plants from Devil’s Point where the contribution of foreign soils across Byers’ sampling sites. However, these ASVs were not shared with other plants being also species-specific.

## Discussion

Despite the relevance of microbial communities in soil and plant health, there are still open questions about the contribution of different biotic and abiotic factors shaping microbial diversity and acquisition in the rhizosphere. A prevailing paradigm is that plant species are one of the most critical factors shaping rhizosphere structure and composition. However, recent studies have shown that plant species alone cannot account for observed patterns. To contribute with new evidence in this area, we carried out the following work using a simplified natural model, the rhizosphere microbiome (bacteria and fungi) of autochthonous vascular plants in Antarctica through 16S rRNA and ITS metabarcoding.

Our results showed that host plant species significantly affect bacterial diversity and richness, and fungal diversity but to a lesser extent. Additionally, the different soils (sampling sites) significantly affect the diversity and species turnover in both fungi and bacteria of the rhizosphere and bulk soil. Finally, the primary source of rhizosphere bacteria seems to be local soils (the same sampling site) with little contribution from the host plant species in acquiring these rhizosphere microbes. On the other hand, acquiring rhizosphere fungi is more complex, being their source local and distant soils across the Byers Peninsula. Here, the host plant species and stochastic processes contribute to the fungal community assembly process in the rhizosphere.

### Microbial diversity differs between plant species, compartments, and sites

This study found that plant species influence rhizosphere microbes’ richness and diversity (phylogenetic and ecological). Here, the rhizosphere of Cq plants harbors richer (Chao1) and more diverse (PD and Shannon) bacterial communities than its counterpart in Da plants. Conversely, fungal communities of Da plant’s rhizosphere were more even (higher Shannon) but neither more phylogenetically diverse nor rich than those of Cq.

Our findings contrast with previous reports for rhizosphere bacteria of Antarctic vascular plants (Da and Cq), where no significant differences in diversity and richness were found between plant species (Teixeira et al., 2010). Unfortunately, no previous studies have compared the diversity of fungi in the rhizosphere of Da and Cq. In contrast, recent studies on other plants (woody plants, understory plants, grasses, and hygrophytic plants) have demonstrated that plant species have a significant influence on the diversity and richness of rhizosphere bacterial communities (Bonito et al., 2014; Ma et al., 2019; Schmid et al., 2019) but limited impact in the rhizosphere’s fungal diversity and richness (Bonito et al., 2014; Xu et al., 2019).

We hypothesize that Da exerts a more substantial rhizosphere effect over the bacterial community than Cq, which explains Da rhizosphere bacteria’s reduced richness and diversity, reflecting the host endemic nature, although both plants are native. This finding agrees with previous evidence that shows that Da plants are better adapted to the harsh conditions of Antarctica, facilitating the establishment of other natives (e.g., Cq) and invasive plants on the continent (Atala et al., 2019). In contrast, we speculate that Da plants offer more microbial niches to rhizosphere fungi than Cq, increasing species’ evenness (higher diversity but equal richness). In woody plants, a decreased frequency of ectomycorrhizal formation is associated with a higher exposed root surface for endophytic fungi to colonize and, consequently, higher levels of fungal diversity (Bonito et al., 2014). To date, there is no evidence of ectomycorrhizal fungi colonization of Da plants, and a large number of endophytic fungi have been isolated from its rhizosphere (de Carvalho et al., 2019 and references therein). Thus, the high abundance of endophytic fungi could explain the higher fungal diversity found in Da’s rhizosphere.

Additionally, we found that different sampling sites and environmental and physicochemical differences between soils (Supplementary Table 2) influenced the diversity (ecological and phylogenetic) and richness of rhizosphere microbial communities. A clear pattern was evident for bacteria, where sampling sites were split consistently into high diversity and richness or low diversity and richness soils. For fungi, this relationship was unclear and did not correlate with bacteria’s diversity or richness or both patterns. In contrast, one previous study did not find significant differences in bacterial communities’ diversity and richness from rhizosphere (Da and Cq) and bulk soils taken at three different sampling sites in King George Island, Antarctica (Teixeira et al., 2010). Despite this, current studies show that on a global scale, the environmental heterogeneity of soil is the primary driver of the diversity and structure of rhizosphere and bulk soil microbial communities (Tedersoo et al., 2014; Fierer, 2017; Yeoh et al., 2017; Ma et al., 2019; Xu et al., 2019, 2; Chu et al., 2020). In our study, we recorded the geographical parameters of the sampling sites, which differ mainly in their altitude and latitude. While we could not determine the rhizosphere and bulk soils’ physicochemical properties, we measured the edaphic properties of 44 rhizosphere and bulk soil samples taken in 2019 at five of the six sampling sites included in this study (Supplementary Table 2). Together, statistically significant (*p*-values ≤ 0.05) edaphic parameters (total C, total N, NO3, Mg, and K) explained the 71.5% (*p*-value = 0.0047) of the variance between sampling sites (Supplementary Figure 6), reinforcing the idea that the combination of geographical and physicochemical factors of the soil defines the sampling sites and directly influences the diversity and richness of microbial communities.

Finally, we found significant differences between compartments where RSS has a higher richness (bacteria and fungi) and diversity (bacteria only) than the rhizosphere. Here, we propose that RSS is a transition compartment between the rhizosphere and bulk soil that support a richer and more diverse (ecologically and phylogenetically) microbial community than the rhizosphere, where both rhizosphere and bulk soil microbial communities coexist. A possible explanation for the observed pattern is the gradient characteristic of the rhizosphere effect, which decreases in its intensity as the distance from the roots increases (Philippot et al., 2013; Fierer, 2017; de la Fuente Cantó et al., 2020; Ling et al., 2022). The rhizosphere effect is weaker in the RSS than in the rhizosphere, allowing the coexistence of microbes that belong to both compartments (rhizosphere and bulk soil).

### Sampling sites and plant species shape the microbial community structure

The main drivers of microbial community composition in both rhizosphere and bulk soil were sampling sites and plant species. The observed differences between communities were more evident when we used a beta diversity distance metric that considered the abundance-weighted phylogenetic composition of ASVs (weighted UniFrac) between communities. The above implies that the different microbial communities harbor distinctive and differentially distributed genetic lineages due to environmental geographic pressures (sampling site, latitude, and altitude) and, to a lesser extent, biotic factors (plant species). In the case of fungi, a more significant fraction of the variance between samples was influenced by other unaccounted variables compared to what we found in bacteria. Altogether, these results are in agreement with what has been described in other studies, where the variance in rhizosphere microbial communities of different plant species is usually more significant if these plants belong to different soils than to plants grown on the same soil (Philippot et al., 2013; Bonito et al., 2014; Yeoh et al., 2017; Xu et al., 2019; Thiergart et al., 2020; Vieira et al., 2020). As we mentioned before, the turnover of fungal species in the rhizosphere and the bulk soil was not determined by the specific soil or the plant species but by other variables that could be relevant yet unmeasured in this study. For example, it has been proposed that the local climatic factors to which the soils are exposed would be the primary driver of the differences between the fungal communities both in the rhizosphere and in the bulk soil (Tedersoo et al., 2014; Thiergart et al., 2020). Due to the contrasting terrain in Antarctica, it is possible to find different microclimates over short distances where factors such as moisture availability, low temperature, and ground-level wind speed significantly influence plants (Beyer et al., 2000). We hypothesize that the high variability at the microclimate level, particularly moisture differences and exposure to sea sprays, could explain the significant differences found in the species turnover of fungi in the rhizosphere and bulk soils presented here.

### The microbial composition of antarctic vascular plants

Our work showed that the microbes associated with the rhizosphere of Antarctic vascular plants and soils were represented mainly by four bacterial and four fungal phyla. Our results agree with those described in the literature where major bacterial groups (i.e., Proteobacteria, Bacteroidetes, Acidobacteria, Planctomycetes, Chloroflexi, and Verrucomicrobia) are widely distributed both in bulk soils and in the rhizosphere of plants throughout the world (Trivedi et al., 2020; Vieira et al., 2020; Ling et al., 2022). Likewise, we found that rhizosphere and bulk soil fungal communities were dominated by Ascomycota, Basidiomycota, Mortierellomycota, and Rozellomycota, which represent cosmopolitan phyla of soil fungi on a global scale (Tedersoo et al., 2014, 2017; Trivedi et al., 2020; Hussain et al., 2021). In brief, our study demonstrates that at higher taxonomic levels (i.e., phylum), the microbes found in bulk soils and plant rhizospheres in Antarctica are ubiquitous to all soils and rhizospheres in the world. Also, the abundance patterns of microbial phyla in the rhizosphere and soil compartments in Antarctica are similar to those described in other environments (Trivedi et al., 2020; Vieira et al., 2020; Ling et al., 2022). For example, Antarctic rhizospheres were enriched in Proteobacteria and Bacteroidetes. In contrast, Acidobacteria, Chloroflexi, Actinobacteria, and Verrucomicrobia were enriched in the surrounding bulk soils. The phyla Proteobacteria and Bacteroidetes thrive in the carbon-rich rhizosphere because of their high metabolic activity, fast growth, and their ability to secrete diverse arrays of carbohydrate-active enzymes that break up the available carbon pool secreted by plant roots (Delgado-Baquerizo et al., 2018; Larsbrink and McKee, 2020; Ling et al., 2022). Finally, the fungal communities of the rhizosphere (especially in Cq) were enriched in the phylum Mortierellomycota compared to the surrounding bulk soil. This phylum increases its dominance at high latitudes and is dominant in tundra biomes (Tedersoo et al., 2014). Also, fungi belonging to this taxa are considered saprotrophs, with some representatives identified as plant growth-promoting fungi (PGPF) that can improve access to the bioavailable forms of P and Fe to the plant (Xu et al., 2019; Ozimek and Hanaka, 2020).

At lower taxonomic levels (genus), we found that the dominant taxa reported in this study (i.e., *Ferruginibacter*, *Candidatus Udaeobacter*, *Polaromonas*, *Sphingomonas*, *Rhodanobacter*, *Gemmatimonas*, *Mucilaginibacter*, *Chthoniobacter*, *Flavobacterium*, and *Bryobacter*) have been reported as relevant bacterial groups in Antarctic soils (Cary et al., 2010; Kim et al., 2012, 2019; Pearce et al., 2012; Guo et al., 2018; Dennis et al., 2019; Lambrechts et al., 2019; Ramírez-Fernández et al., 2019; Almela et al., 2021; Marcoleta et al., 2022). Likewise, we found that some abundant genera of fungi reported in this work (i.e., *Mortierella*, *Glaciozyma*, *Antarctomyces, Mrakia*, *Pseudogymnoascus*, *Herpotrichia*, *Juncaceicola*, *Microdochium*, *Leptosphaeria*, and *Dioszegia*) have also been described as essential components of Antarctic soils (Pearce et al., 2012; Aislabie et al., 2014; de Andrade et al., 2018; Firdaus-Raih et al., 2018; de Carvalho et al., 2019; Garrido-Benavent et al., 2020; Horrocks et al., 2020). Also, at the genus level, we were able to identify microbial taxa that were significantly enriched in the plant-associated compartments (rhizosphere and RSS). Some of these bacterial genera have been described as essential parts of the rhizosphere microbiome in woody plants and grasses in other environments globally. For example, the genera *Dyadobacter* (diazotrophic bacteria), *Mucilaginibacter, Asticcacaulis*, and *Neorhizobium* (diazotrophic bacteria) are considered plant growth-promoting bacteria (PGPB) and are part of the core microbiome of diverse plants and cultivars across the globe (Costa et al., 2006; Madhaiyan et al., 2010; Wei et al., 2017; Yeoh et al., 2017; Ishizawa et al., 2018; Xu et al., 2018; Liu et al., 2019; You et al., 2021). Strains of *Devosia* have been isolated from the rhizosphere of Cq and display a high tolerance to salt, where *in vitro* experiments have demonstrated that this bacteria can improve the osmotic and physiological performance of the Antarctic vascular plants (Gallardo-Cerda et al., 2018). Additionally, this genus is globally abundant in soil and has been found enriched in the rhizospheres of various plants of economic interest (Delgado-Baquerizo et al., 2018; Wang et al., 2022). Finally, representative genus *Galbitalea* has not been obtained from vascular plant rhizospheres; however, several isolates have been obtained from Antarctic lichens (Noh et al., 2021).

Similar results were obtained for fungi, where genera such as *Dioszegia*, *Leptosphaeria*, *Verrucaria*, and *Microdochium* were significantly enriched in the rhizosphere and RSS of some Antarctic vascular plants. Fungi from *Dioszegia* and *Microdochium* are part of the core microbiome in various plants’ rhizospheres, where they play an essential role in controlling and modulating the composition and diversity of plants’ bacterial communities and pathogenic bacteria (Douglas and Deacon, 1994; Agler et al., 2016; Gómez Expósito et al., 2017; Pascale et al., 2020; Schlatter et al., 2020). Other genera such as *Leptosphaeria* are usually classified as pathogenic fungi; however, they are core taxa in different plant species and are defined as dark septate endophytic fungi (DSE) (Verma et al., 2017; Xu et al., 2019; Fortin Faubert et al., 2022). Also, some of their representatives have a beneficial impact on the plants that grow through the secretion of indoleacetic acid and siderophores (Verma et al., 2017; Xu et al., 2019; Fortin Faubert et al., 2022). Finally, fungi from the genus *Verrucaria* are known taxa of lichenized fungi, widely described in Antarctic soils (Lachacz et al., 2018; Garrido-Benavent et al., 2020), but their presence in rhizospheres of Antarctic vascular plants has not been reported before.

### The acquisition of rhizosphere microbes in antarctic vascular plants

One of our main goals was to quantitatively determine the possible source of microbes in the rhizosphere of Antarctic vascular plants. As in other rhizospheres, we considered two plausible hypotheses explaining the source of the microbes that make up the rhizosphere microbiome in Da and Cq. One hypothesis considers that the different soils harbor specific groups of microbes, which represent the total pool that the root can recruit. Then, the secretion of root exudates alters the chemical composition of the surrounding soil, serving as substrates for the growth of selected soil microbes. Here, the soil would be the primary source of microbial communities in the rhizosphere. A second hypothesis considers that the rhizosphere microbial communities are partially inherited through the vertical transmission of microbes from the seed or other propagule types. Thus, the plant species would have a central role in assembling the microbial communities of the rhizosphere, somewhat independently of soil microbial composition.

Our results support the first hypothesis for bacteria, where the rhizosphere bacterial communities are acquired mainly from local soil sources, and plant species have little incidence in the specificity of the bacterial community composition in the rhizosphere. In contrast, our results are not conclusive for fungi, showing that local (Da and Da.Cq) and more distant soils (Cq) are important sources for acquiring these microbes but are not the determining factor, especially in and Da.Cq rhizospheres. Likewise, plant species were also a relevant factor (mostly in Cq plants) in recruiting specific fungal ASVs to the rhizosphere, but other stochastic factors would contribute significantly to the assembly process of rhizosphere fungal communities. Additionally, coastal Antarctic soils are heavily influenced by organic inputs of seabirds and marine vertebrates’ feces that facilitate the establishment of vascular plants and could contribute as sources of microorganisms, as well as their spread (Ramírez-Fernández et al., 2019). Studies have shown that forces such as long-range transport through wind and ocean currents might also play a role (Archer et al., 2019).

In general, our results regarding the source and acquisition of the rhizosphere bacteriome in Antarctic plants agree with multiple experiments conducted in common garden setups, where the soil is the primary source for the acquisition of rhizosphere bacteria (Schlaeppi et al., 2014; Fitzpatrick et al., 2018; Leff et al., 2018; Hannula et al., 2019; Thiergart et al., 2020). Likewise, these experiments also showed that in the case of fungi, the effect of the host species has a stronger force in the acquisition of the fungal communities in the rhizosphere from soil (Hannula et al., 2019; Thiergart et al., 2020). It is important to note that in the case of fungi, various studies point to the existence of stochastic factors that randomly determine the assembly of fungal communities in the rhizosphere, the main one being the dispersal ability of fungi in the soil (Hussain et al., 2021; Zhang et al., 2021).

Taken together, we demonstrate the usefulness of Antarctic vascular plants as a model for the study of plant–microbe interactions in a natural environment without the noise of other more accessible environments.

## Conclusion

The present work represents the most recent and complete study of microbial ecology from Antarctic vascular plant rhizospheres, contributing to disentangling the interactions that shape the diversity and structure of microbial communities. Here, we show that the diversity and composition of rhizosphere microbial communities in Antarctic plants are determined by the host species identity and the specific sampling site. This last factor is also the main driver of microbial species turnover in conjunction with geographic factors such as latitude and altitude. Moreover, we show that the acquisition of the rhizosphere bacteria is given by the pool of bacteria available in the local soil, where the influence of the host plant species is minimal. In contrast, the acquisition of rhizosphere fungal communities is influenced by the interaction of local and distant soils, host plant species, and stochastic factors that produce a more random assemblage of fungal species.

## Data availability statement

The datasets presented in this study can be found in online repositories. The names of the repository/repositories and accession number(s) can be found in the article/Supplementary material.

## Author contributions

EC-N, KC, MM-M, and FG conceived the study. EC-N, FG, and JG-C conducted the fieldwork. FG, JG-C, and SG-L processed the samples. SG-L, JA, and EC-N analyzed the data. SG-L and EC-N wrote the manuscript. IA-R, MP-L, SG-L, and EC-N revised previous versions of the manuscript. All authors contributed and agreed on the final version of the manuscript.

## Footnotes

1
www.github.com/twbattaglia/btools
