# Supplementary material for: Source and acquisition of rhizosphere microbes in Antarctic vascular plants
Source: Front Microbiol. 2022 Sep 8;13:916210. doi: 10.3389/fmicb.2022.916210 (PMC9493328; doi:10.3389/fmicb.2022.916210)
Supplement: Supplementary file 2 [file Data_Sheet_1.PDF]

Origin and acquisition of rhizosphere microbes in Antarctic vascular plants

Sergio Guajardo-Leiva; Jaime Alarcón; Florence Gutzwiller; Jorge Gallardo-Cerda; Ian S. Acuña-Rodríguez; Marco Molina-Montenegro; Keith A. Crandall; Marcos Pérez-Losada and Eduardo Castro-Nallar.

Supplementary Figures

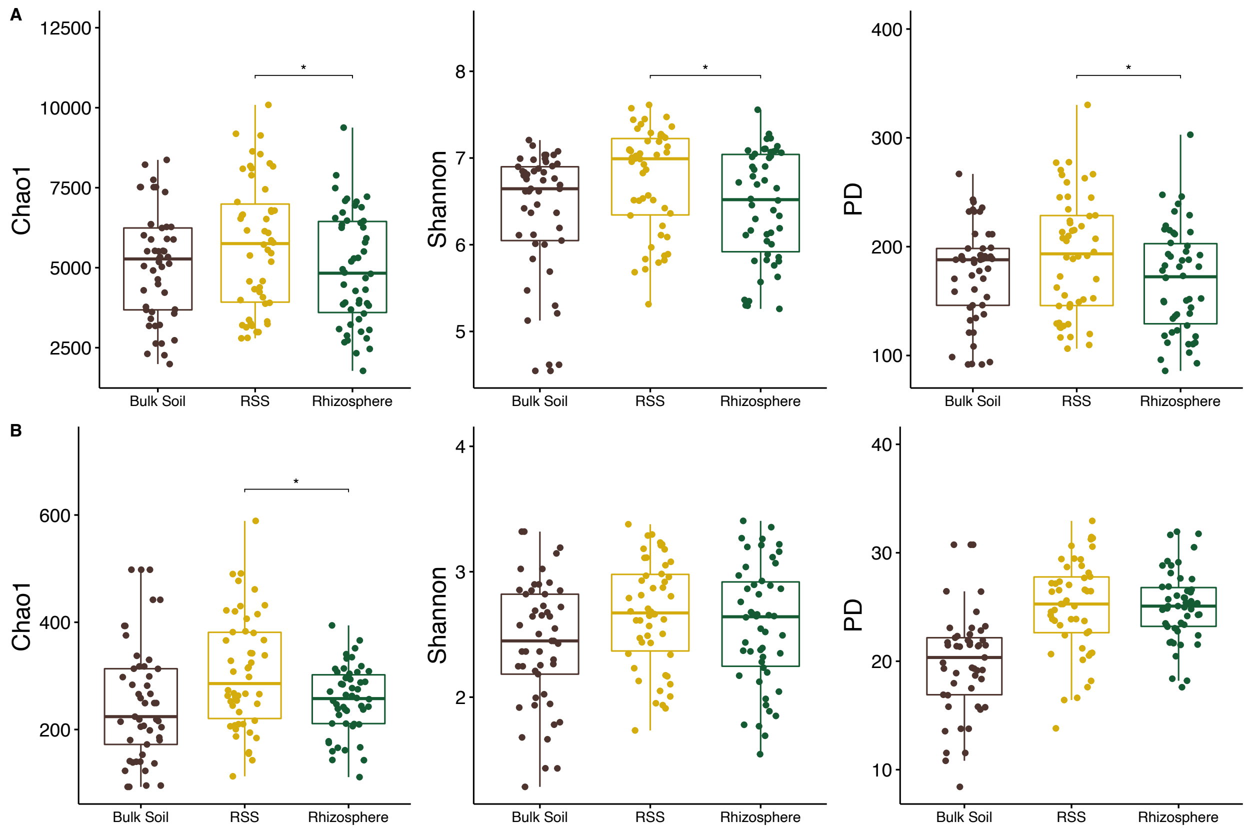

Supplementary Figure S1. Bacteria (A) and fungi (B) alpha diversity (Chao1; Shannon; Phylogenetic Diversity) for compartments (rhizosphere, rhizosphere surrounding soil [RSS], and Bulk Soil). Dots represent data points, and boxplots represent the interquartile range of alpha diversity. Statistically significant differences (Tukey`s HSD p-value ≤ 0.05, for predicted alpha diversity by a Linear Model) are marked by brackets.

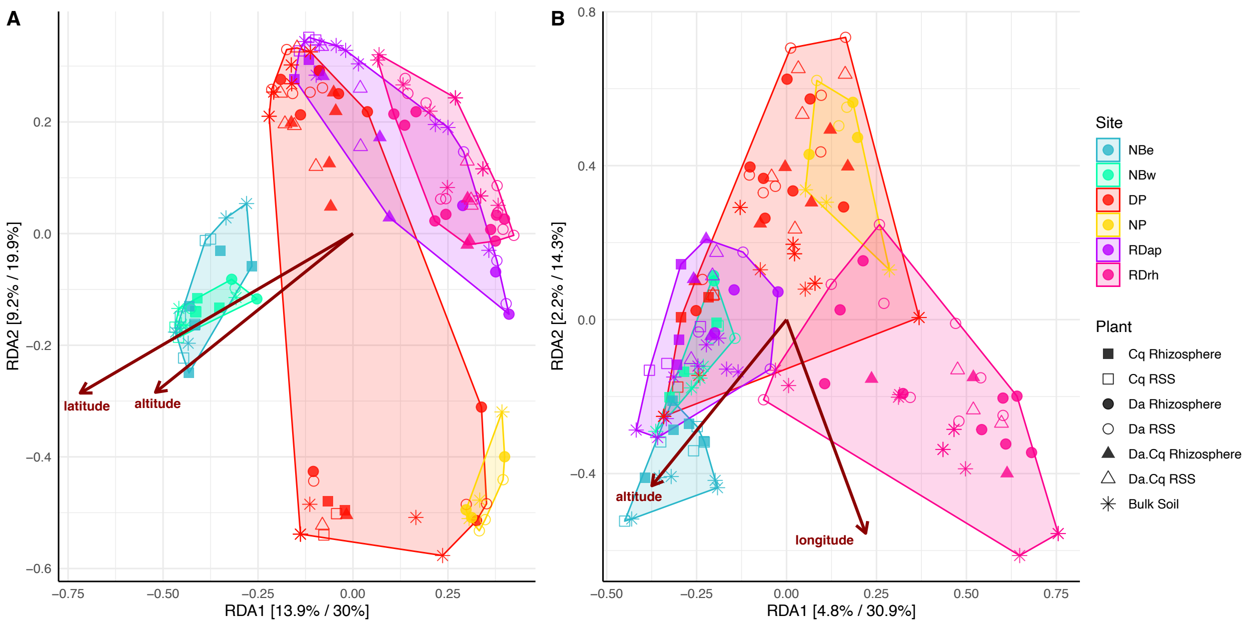

Supplementary Figure S2. Bacteria (A) and fungi (B) beta-diversity for *Deschampsia antarctica* [Da], *Colobanthus quitensis* [Cq], and their association [Da.Cq]. A Redundancy Analysis of Hellinger transformed Bray-Curtis distance was statistically chosen to describe microbial community structure in a supervised approach. Each axis shows the percentage of variance explained in an unsupervised and supervised analysis.

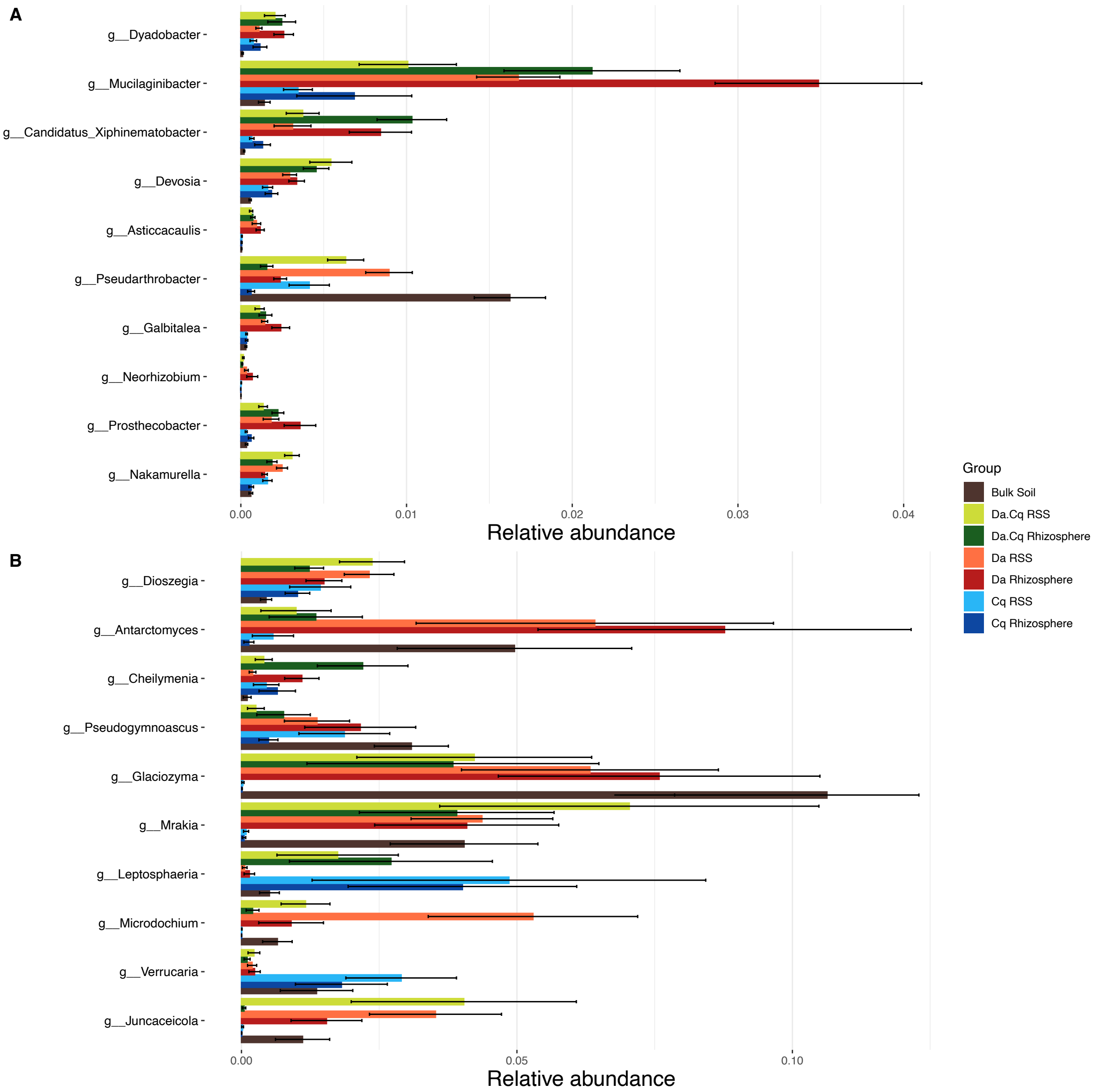

Supplementary Figure S3. Bacteria (A) and fungi (B) taxonomic markers of bulk soil and plant's rhizospheres and RSS from *Deschampsia antarctica* [Da], *Colobanthus quitensis* [Cq], and their association [Da.Cq]. Bars represent the arithmetic mean of taxa abundance in each compartment and error bars the standard deviation. Taxa in the Y-axis are ordered according to their importance (mean decrease in Gini coefficient) in the model

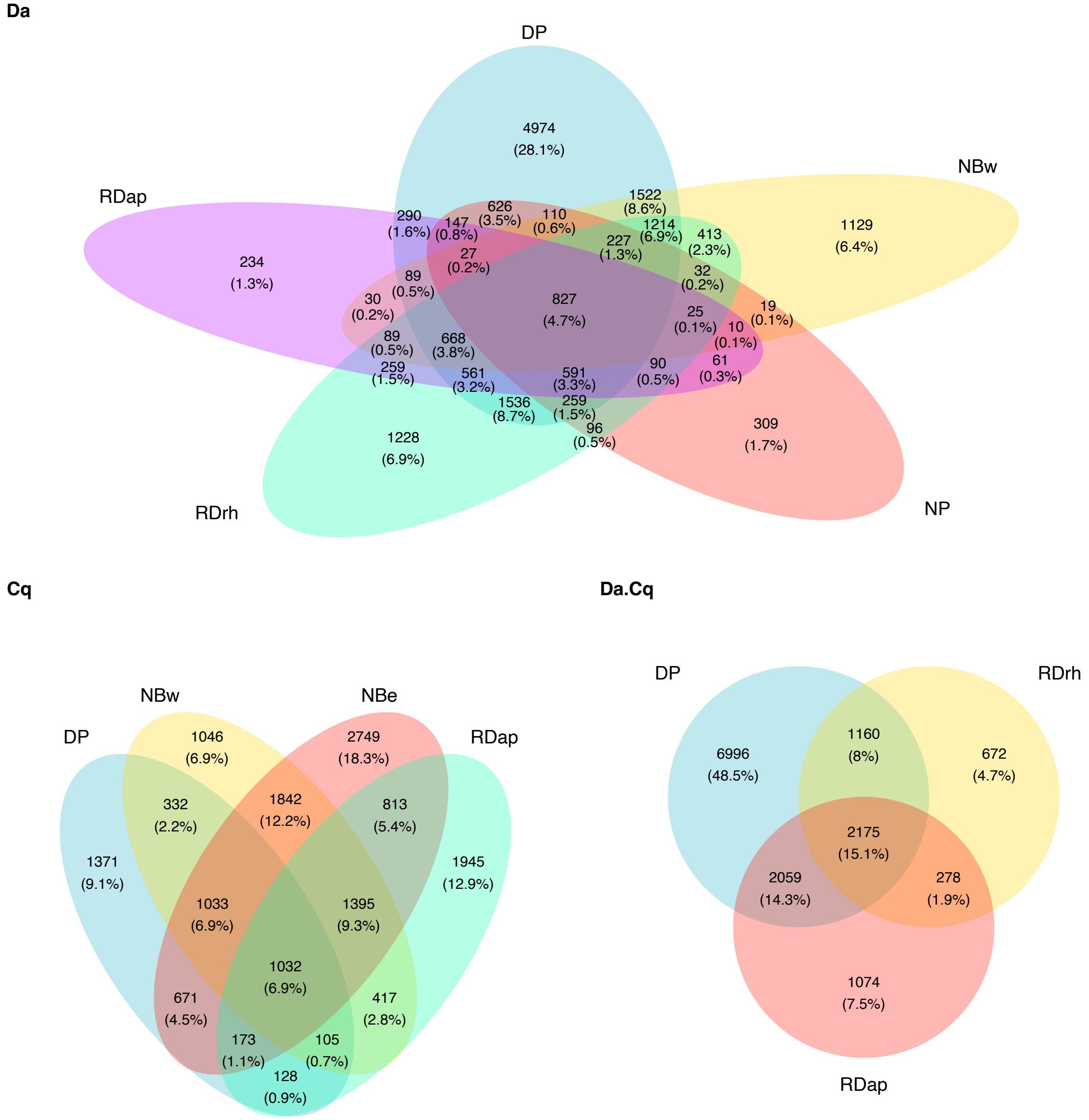

Supplementary Figure S4. Core microbiome analysis of Bacteria from rhizospheres of *Deschampsia antarctica* [Da], *Colobanthus quitensis* [Cq], and their association [Da.Cq]. Integers in Venn diagrams represent the number of shared and exclusive ASVs between different sampling sites (and intersections) where the plant´s rhizospheres were collected. Percentages between parenthesis represent the relative abundance of the ASVs sets and intersections.

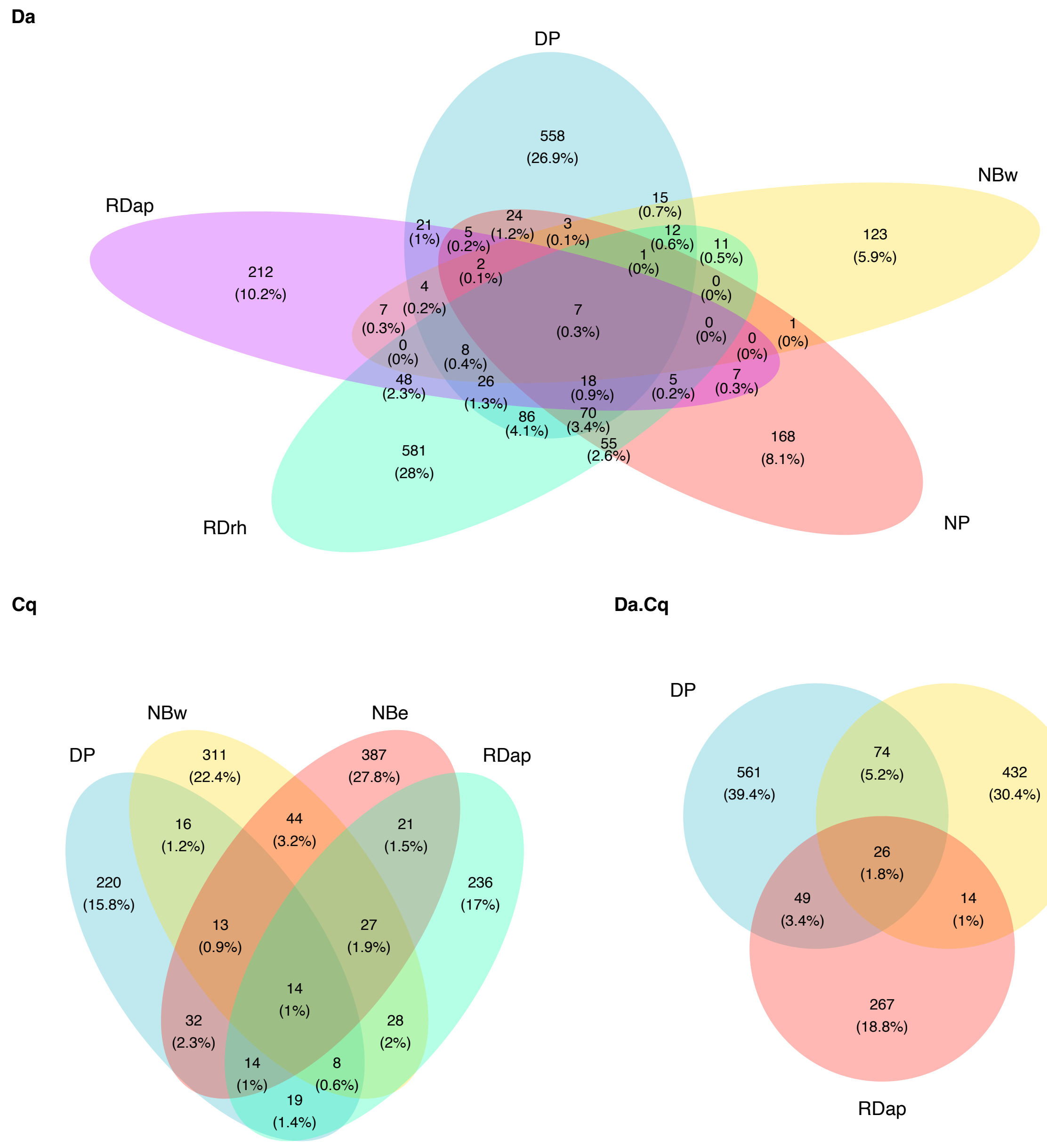

Supplementary Figure S5. Core microbiome analysis of Fungi from rhizospheres of *Deschampsia antarctica* [Da], *Colobanthus quitensis* [Cq], and their association [Da.Cq]. Integers in Venn diagrams represent the number of shared and exclusive ASVs between different sampling sites (and intersections) where the plant´s rhizospheres were collected. Percentages between parenthesis represent the relative abundance of the ASVs sets and intersections.

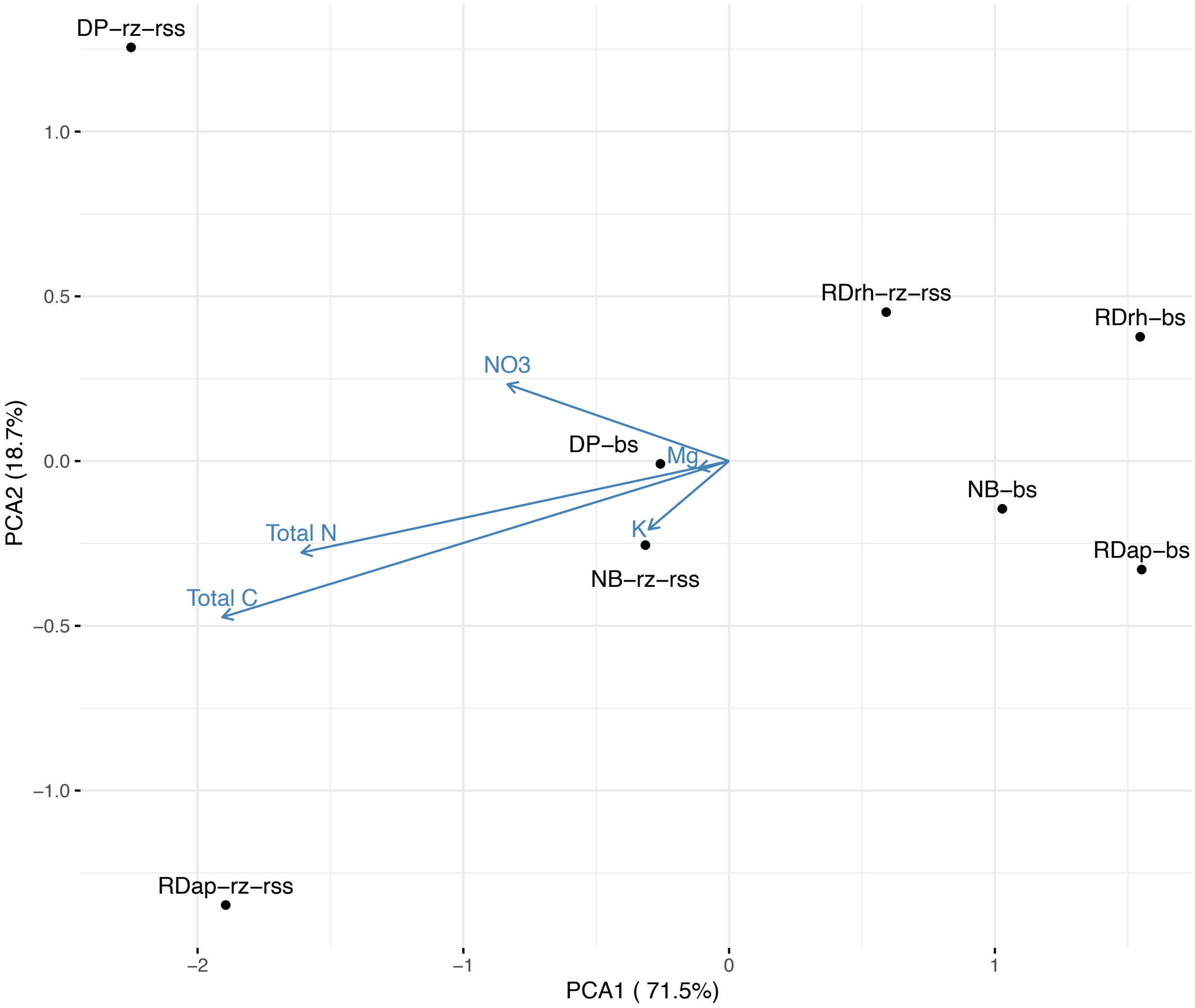

Supplementary Figure S6. Principal component analysis (PCA) of sampling sites based on the edaphic properties of Rhizosphere-RSS [rz-rss] and bulk soils [bs]. Soil samples were obtained at North Beach [NB], Devil`s Point [DP], Rotch Dome Ritli Hill [RDrh], and Rotch Dome Amadok Point [RDap] in February 2019, a year after the microbiological sampling at the same sites. Only statistically significant (p-value ≤ 0.05) edaphic properties were plotted. PCA 1 is statistically significant (p-value $= 0.0047$) and accounts for 71.5% of the total variation.
